# Effect of COVID-19 on sexual function and activities among reproductive-age women in Ibadan, South-West Nigeria

**DOI:** 10.1093/sexmed/qfae004

**Published:** 2024-02-21

**Authors:** Rukiyat Adeola Abdus-Salam, Oluwasegun Caleb Idowu, Fatimat Motunrayo Akinlusi, Yusuf Bello, Imran Oludare Morhason-Bello

**Affiliations:** Department of Obstetrics and Gynaecology, University College Hospital, Ibadan 200212, Nigeria; Department of Obstetrics and Gynecology, College of Medicine, University of Ibadan, Ibadan 200005, Nigeria; Department of Obstetrics and Gynaecology, University College Hospital, Ibadan 200212, Nigeria; Department of Obstetrics and Gynecology, Lagos State University College of Medicine/Lagos State University Teaching Hospital, Ikeja 100254, Nigeria; Department of Statistics, Faculty of Science, University of Ibadan, Ibadan 200005, Nigeria; Institute of Advanced Medical Research and Training, College of Medicine, University of Ibadan, Ibadan 200212, Nigeria; Department of Obstetrics and Gynaecology, University College Hospital, Ibadan 200212, Nigeria; Department of Obstetrics and Gynecology, College of Medicine, University of Ibadan, Ibadan 200005, Nigeria; Institute of Advanced Medical Research and Training, College of Medicine, University of Ibadan, Ibadan 200212, Nigeria

**Keywords:** COVID-19 effects, COVID-19 sexual dysfunction, female sexual dysfunction, COVID-19 sexual activities, COVID-19 pandemic, COVID-19 lockdown sexual dysfunction

## Abstract

**Background:**

Since the outbreak of COVID-19 disease, the environment, families, individuals, and their ways of living have been affected. Social distancing was one of the strategies for the prevention of SARS-CoV-2 infection. It led to limited social interactions for fear of contracting the infection, which ultimately affected relationships, among which is sexual health.

**Aim:**

To determine the risk of female sexual dysfunction and the effect of the COVID-19 pandemic on sexual function and activities among women of reproductive age in Ibadan, South-West Nigeria.

**Methods:**

This cross-sectional study of 218 reproductive-age women evaluated the sexual function before, during, and after the COVID-19 disease pandemic and lockdown. Pretested semistructured self-administered questionnaires with the Female Sexual Function Index (FSFI) were used for data collection. Information collected included sociodemographic and gynecologic characteristics and COVID-19 experiences, as well as sexual history and function before, during, and after the COVID-19 pandemic lockdown. The level of significance was set at *P* < .05.

**Outcomes:**

Respondents were aged 20 to 50 years (95%) with a mean ± SD age of 34.82 ± 8.52 years; the majority were married (75.58%); one-fifth (21.9%) tested positive for COVID-19 infection; and participants who tested positive for COVID-19 infections were mostly health care workers.

**Results:**

An absence of sexual activity was reported in 9.18% of participants during lockdown, 7.73% before lockdown, and 4.18% after lockdown. The risk of female sexual dysfunction was prevalent among participants, especially those who tested positive for COVID-19 infection. The prevalence was worse during the pandemic lockdown (53.57%) than before (48.39%) or after (51.61%), and a similar pattern was seen among those who tested negative. There was no statistically significant difference in risk of developing sexual dysfunction during the COVID-19 pandemic between those who tested positive and negative to COVID-19. The arousal and desire domains contributed the highest proportion of low FSFI scores.

**Clinical Implications:**

Nationwide lockdowns, which may be a method of control for pandemics, may result in psychosocial complications such as female sexual dysfunction.

**Strength and Limitations:**

Most respondents had tertiary education and were able to respond to self-administered questionnaires, yet the risk of recall bias remains a concern whereas the pandemic met the world unprepared and baseline FSFI prior the pandemic was not available for participants. There are no local validation studies for the FSFI in Nigeria, which could have aided interpretation of results.

**Conclusion:**

A low FSFI score is prevalent in Ibadan, South-West Nigeria, with a higher incidence reported during the COVID-19 pandemic lockdown.

## Introduction

The novel coronavirus (SARS-CoV-2) causing the coronavirus disease (COVID-19) was first reported in Wuhan, China, in 2019 and soon became a global pandemic in 2020, with severe health and economic consequences forcing nations to declare nationwide lockdown to limit the ravaging pandemic, which resulted in further socioeconomic impacts.^1–5^

The COVID-19 pandemic affected the daily life of individuals and was associated with increased risk of depression and anxiety.^6–9^ Limited social interaction and the risk of contracting the infection affected the lives of individuals, families, and relationships, including sexual health. Female sexual dysfunction (FSD) was often underreported, and it remains globally prevalent and may be worse in developing nations such as Nigeria even before COVID-19 pandemic.^10–14^ The pandemic, social distance, and associated depression and anxiety may result in worsening sexual dysfunction.

With COVID-19 being a novel infection, much is unknown about the effect of the infection on the sexual function of women. The duration of the lockdown experienced globally was also of unprecedented proportion; the effect on sexuality and its determinants was largely unknown. Studies in Turkey and Poland showed that the frequency of sexual intercourse and the level of sexual function were lower during the pandemic among men and women, with reduced Female Sexual Function Index (FSFI) scores.^15^^,^^16^ In studies in Italy and the United States, similar findings were reported, with a reduction in each of the domains across the FSFI.^17–19^

What is the current level of risk of sexual dysfunction among women in Ibadan, South-West Nigeria? Did COVID-19 infection and lockdown reduce the frequency of sexual activity or increase the risk of FSD among this population of women?

## Methods

This cross-sectional study evaluated the sexual function of women before, during, and after the COVID-19 pandemic in Ibadan. It was conducted among sexually active women over a 3-month period at the outpatient clinics of the University College Hospital, Ibadan. The study population included young and middle-aged women who were sexually active. The eligibility criteria included sexually active, apparently healthy women older than 18 years. Nonconsenting women, women with illness requiring hospital admission, and women with severe systemic disease or mental illness were excluded. Minimum sample size of 196 is required for this cross-sectional study of a single population, with a 95% confidence level based on a prevalence rate of 85% from a previous study in Ibadan^10^ and a power of 80%. A total of 218 women were enrolled into the study, making room for a 10% attrition rate. A nonprobability purposive sampling technique was used for patient selection. A pretested semistructured self-administered questionnaire was administered in a private environment. Data were imputed and analyzed with SPSS version 23.0 (IBM). Descriptive univariate analysis, bivariate analysis, and multiple logistic regression were done. The level of significance was set at 5%. Ethical approval was obtained from the University of Ibadan/University College Hospital ethics committee (UI/UCH/21/0073).

### FSFI questionnaire

The FSFI is a standardized 19-item questionnaire with responses designed on a Likert scale (score, 0-5); the higher the score, the better the response. These 19 questions were distributed across the domains of female sexual function, making it possible to derive a cumulative score for each of the 6 domains. The summary of the FSFI used is shown in Table A1 with cutoff scores based on the Sahar classification for mild dysfunction.^20^ FSFI has not been validated for women in Ibadan, South-West Nigeria, but studies across Nigeria have used the FSFI to demonstrate sexual dysfunction among Nigerian women.^10^^,^^21^^,^^22^ In the presence of distress, an FSFI score below the cutoff may be regarded as sexual dysfunction; a low FSFI score has been demonstrated to reflect risk of sexual dysfunction.^17^ As such, FSFI scores below the cutoff are regarded as a risk of developing FSD.

**Table A1:** Cutoff distribution for mild sexual dysfunction by Female Sexual Function Index according to Sahar classification.

	**Item**		**Score**		
**Domain**	**Nos.**	**Sum range**	**Factor**	**Range**	**Cutoff**
Desire	1, 2	10	0.6	1.0–6.0	3.80
Arousal	3, 4, 5, 6	20	0.3	0–6.0	4.07
Lubrication	7, 8, 9, 10	20	0.3	0–6.0	4.09
Orgasm	11, 12, 13	15	0.4	0–6.0	4.23
Satisfaction	14, 15, 16	15	0.4	1.0–6.0	4.78
Pain	17, 18, 19	15	0.4	0–6.0	4.07
Total	1-19	95	—	2–36	26.5

## Results

A total of 218 participants were enrolled into the study. Table 1 shows descriptive statistics of the sociodemographic characteristics of the participants. The participants were aged 18 to 59 years, with a mean ± SD age of 34.82 ± 8.52 years. All participants were Nigerians: the majority were Yoruba (*n* = 166, 76.15%), 12.8% were Ibos, 1.4% were Hausas, and 21 (9.63%) were from tribes other than the 3 major Nigerian tribes. The majority of participants were married (*n* = 164, 75.58%); about a quarter were single (*n* = 50, 23.14%); and the others were either divorced or widowed. Over 90% had tertiary education and the rest had secondary education.

**Table 1:** Descriptive statistics of participants’ demographic characteristics.

	**COVID-19 status, No. (%)**			
**Variable**	**Total** ^**a**^	**Positive (*n* = 36)**	**Negative (*n* = 54)**	***P* value**
Age, y, mean (SD)	34.82 (8.53)	36.67 (1.38)	34.80 (1.34)	.348
Occupation				.001
Health care worker	76 (35.02)	29 (80.56)	24 (46.15)	
Others	129 (59.45)	7 (19.44)	28 (53.85)	
Ethnicity				.394
Yoruba	166 (76.15)	25 (69.44)	42 (77.78)	
Igbo	28 (12.84)	5 (13.89)	7 (12.96)	
Hausa	3 (1.38)	2 (5.56)	0 (0.0)	
Others	21 (9.63)	4 (11.11)	5 (9.26)	
Education				.272
Pretertiary	18 (8.29)	0	3 (5.56)	
Tertiary	199 (91.71)	36 (100)	51 (94.44)	
Marital status				.882
Single	50 (23.04)	8 (22.22)	14 (25.93)	
Married	164 (75.58)	28 (77.78)	39 (72.22)	
Others	4(1.83)	0	1 (1.85)	

^a^Total includes participants without COVID-19 test and inconclusive results.

The participants were grouped by prior exposure to COVID-19: 36 (21.95%) tested positive for COVID-19 infection, whereas 54 (32.93%) tested negative. Nearly half the participants did not take the COVID-19 test (*n* = 72, 43.90%), and a couple of the participants’ test results were inconclusive (*n* = 2, 1.22%). The occupation of the participants (categorized as health care workers or others) was significantly associated with the COVID-19 test outcome (χ^2^[1] = 10.51, *P* = .001); that is, health care workers were more likely to test positive. There was no significant difference in the mean age of participants between the groups with positive and negative test results (*t*[85] = 0.94, *P* = .35).

More participants reported not engaging in sexual activity during the lockdown (9.18%) than before the lockdown (7.73%) and after the lockdown (4.81%). Among those who engaged in sexual activities, FSD was more prevalent in women who tested positive for COVID-19 infection during the pandemic (53.57%) vs before (48.39%) or after (51.61%) the pandemic. FSD was also more common during the COVID-19 pandemic among women who tested negative for the virus (69.81%) when compared with women who tested positive (Table 2).

**Table 2:** Risk prevalence of female sexual dysfunction before, during, and after the COVID-19 pandemic lockdown.

	**COVID-19 status**					
	**Total (*N* = 218)**		**Positive (*n* = 36)**		**Negative (*n* = 54)**	
**Period**	**No.**	**Prevalence, % (95% CI)**	**No.**	**Prevalence, % (95% CI)**	**No.**	**Prevalence, % (95% CI)**
Before^a^	121/191	63.35 (56.23–69.94)	15/31	48.39 (31.19–65.97)	35/53	66.04 (52.06–77.68)
During^b^	119/188	63.30 (56.11–69.94)	15/28	53.57 (34.87–71.32)	37/53	69.81 (55.90–80.84)
After^c^	114/198	57.58 (50.53–64.33)	16/31	51.61 (34.03–68.81)	32/53	60.38 (46.44–72.81)

^a^Nonresponse, *n* = 11. No sexual activities before the COVID-19 pandemic, *n* = 16 (positive, *n* = 4; negative, *n* = 1).

^b^Nonresponse, *n* = 11. No sexual activities during the COVID-19 pandemic, *n* = 19 (positive, *n* = 7; negative, *n* = 1).

^c^Nonresponse, *n* = 10. No sexual activities after the COVID-19 pandemic, *n* = 10 (positive, *n* = 4; negative, *n* = 1).

Table 3 shows the pattern of risk of FSD after the COVID-19 pandemic by the participants’ sociodemographic characteristics. Following the COVID-19 pandemic, married women who tested positive had a higher prevalence of FSD (93.75%) than those who tested negative (65.63%).

**Table 3:** Prevalence of female sexual dysfunction after the COVID-19 pandemic lockdown by participants’ demographic characteristics.

	**COVID-19 status**					
	**Total** ^**a**^		**Positive** ^**b**^ **(*n* = 36)**		**Negative** ^**c**^ **(*n* = 54)**	
**Variable**	**No.**	**Prevalence, % (95% CI)**	**No.**	**Prevalence, % (95% CI)**	**No.**	**Prevalence, % (95% CI)**
Occupation	108		16		31	
Health care worker	34	31.48 (23.36–40.93)	13	81.25 (52.88–94.36)	13	41.94 (25.57–60.29)
Others	74	68.52 (59.07–76.64)	3	18.75 (5.64–47.12)	18	58.06 (39.71–74.43)
Ethnicity	114		16		32	
Yoruba	91	79.82 (71.36–86.27)	12	75.00 (47.02–91.02)	25	78.13 (59.90–89.52)
Igbo	9	7.89 (4.13–14.57)	1	6.25 (0.75–37.06)	4	12.50 (4.58–29.81)
Hausa	2	1.75 (0.43–6.84)	1	6.25 (0.75–37.06)	0	0
Others	12	10.53 (6.04 -17.72)	2	12.50 (2.82–41.30)	3	9.38 (2.92–26.27)
Education	114					
Pretertiary	13	11.40 (6.70–18.75)	0	0	2/30	6.25 (1.48–22.81)
Tertiary	101	88.60 (81.25–93.30)	100	100	30/32	93.75 (77.19–98.52)
Marital status	114		16		32	
Single	24	21.05 (14.47–29.59)	1	6.25 (0.75–37.06)	10	31.25 (17.28–49.73)
Married	88	77.19 (68.51–84.04)	15	93.75 (62.94–99.25)	21	65.63 (47.20–80.30)
Others	2	1.75 (0.43–6.84)	0	0	1	3.13 (0.40–20.38)

^a^Nonresponse, *n* = 10. No sexual activities after COVID-19 to the time of the study, *n* = 10.

^b^Nonresponse, *n* = 1. No sexual activities after COVID-19 to the time of the study, *n* = 4.

^c^No sexual activities after COVID-19 to the time of the study, *n* = 1.

**Figure 1:**
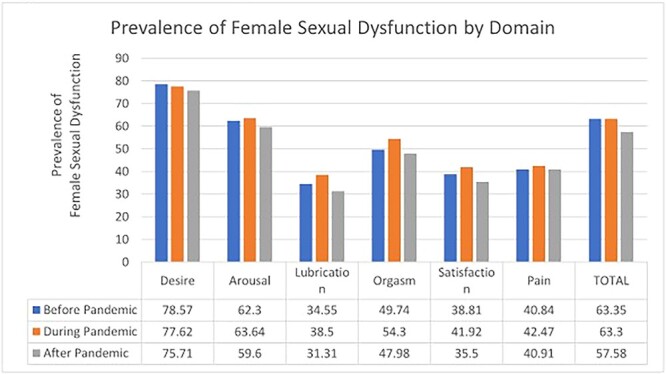
Risk prevalence of female sexual dysfunction by domain.

The average FSFI score was lowest during the pandemic and highest after the lockdown. The average score was also lowest during COVID-19 in all the domains of sexual function tested, except lubrication and pain disorders. The distribution of FSD by domain was examined per the Sahar classification, which uses a higher cutoff for each domain (Table A1).^20^ The prevalence of FSD is shown in Figure 1 by domain. Except for desire, the COVID-19 pandemic period had the highest prevalence of FSD in each domain. In Table 4, mean FSFI scores were compared before, during, and after the pandemic by analysis of variance, which showed no significant difference (*F*_2, 541_ = 2.36, *P* = .46).

**Table 4:** Female Sexual Function Index scores before, during, and after the COVID-19 pandemic lockdown.

**Period**	**Score, mean (SD)**	***P* value**
COVID-19		.063
Before	22.71 (7.70)	
During	21.92 (8.36)	
After	23.69 (6.79)	

Due to the high prevalence of sexual dysfunction risk among the women following the COVID-19 pandemic, Poisson regression was used to investigate the association between the prevalence of sexual dysfunction risk and COVID-19 test outcome (Table 5). First, the model was built with the COVID-19 test outcome as the primary dependent variable, while the participants’ demographic characteristics were adjusted for in the second model. With a crude risk ratio of 1.03 (95% CI, 0.89–1.19), women who tested positive for COVID-19 were 1.03 times more likely to develop FSD than those who tested negative for the virus. The risk of FSD among participants who tested positive for COVID-19 decreased when the sociodemographic characteristics were controlled for in the model (adjusted risk ratio, 0.98; 95% CI, 0.83–1.16). These results showed that the sociodemographic characteristics of the women significantly changed the impact of COVID-19 infection on FSD.

**Table 5:** Poisson regression of the effect COVID-19 infection on female sexual dysfunction.

	**Risk ratio (95% CI)**		
**Variable**	**Crude**	**Adjusted**	***P* value**
COVID-19 infection			.797
Negative	1 [Reference]	1 [Reference]	
Positive	1.03 (0.89–1.19)	0.98 (0.83–1.16)	
Sociodemographic variable: age		1.00 (0.98–1.01)	
Occupation			.354
Other		1 [Reference]	
Health care worker		1.09 (0.91–1.30)	
Ethnicity			.572
Others		1 [Reference]	
Yoruba		0.95 (0.74–1.23)	
Igbo		1.09 (0.81–1.45)	
Hausa		1.07 (0.63–1.82)	
Education			.912
Pretertiary		1 [Reference]	
Tertiary		1.03 (0.66–1.60)	
Marital status			.013
Single		1 [Reference]	
Married		1.20 (0.95–1.52)	
Others		0.71 (0.53–0.95)	

## Discussion

This study assessed the risk of FSD before, during, and after COVID-19 pandemic lockdown in Nigeria using the FSFI. The study showed that more women were not engaging in sexual activities during the lockdown period as compared with before and after the lockdown. There was a relatively higher prevalence of risk of FSD among those who reported sexual activities during the lockdown than before and after the period.

The reduction in frequency of sexual activities during COVID-19 and the high incidence of FSD among those who had sexual activities are similar to other studies, which have been associated with the fear of the unknown about COVID-19 during the lockdown period. During this period, there was general tension and pervasive fear from acquiring “a disease that has no cure.” This study was conducted at a period when the vaccine was not available in Nigeria and fatality from the COVID-19 was high. It is also plausible that the reduction in sexual activity might be due to the overwhelming economic hardship, restriction in movement, and limited physical activities and social interactions, among others.^23^

There appeared to be a slight reduction in the FSFI score during the lockdown. Mean FSFI scores were lowest during the pandemic/lockdown. Several studies reported reduction in sexual function among men and women globally with reasons such as reduced physical activities, psychosocial distress, economic hardships, and movement restrictions.^15^^,^^17^^,^^19^^,^^23^ In each domain of sexual function, the risk prevalence of developing FSD (at least mild) was highest during the pandemic/lockdown as compared with other periods; the only exception to this was the desire domain. This may imply that participants still had a desire for sexual intercourse but their activity and function may have been lowered by the reduced social interaction.

However, no relationship has been established between testing positive to COVID-19 infection and FSD. Women who tested positive for COVID-19 were 1.03 times more likely than those who tested negative for the virus to develop FSD, but this was not statistically significant. Although this study suggests that there may be some cause-and-effect relationship between COVID-19 pandemic lockdown and FSD, it will require well-structured studies to clearly demonstrate such relationships. It appears that the reduction in social interaction during the pandemic, rather than the COVID-19 infection, resulted in the reduction in sexual function.

There are no locally validated cutoff scores for testing sexual dysfunction across each domain in Nigeria; as such, the Sahar classification, which was validated among Egyptian women, was used.^20^ The cutoff levels to consider dysfunction among women in Ibadan may be different, and this may be a limitation to the interpretation of the FSFI scores, especially in the individual domains. However, the inference of sexual dysfunction based on total FSFI score seems to be relatively uniform across studies using the FSFI to check for sexual dysfunction.^20^^,^^24^ There is a need for local studies to validate the FSFI and give cutoffs for sexual dysfunction across each domain applicable to women in Nigeria.

Another limitation to the study was the reliance on participants’ ability to recall their sexual activities and function before and during the COVID-19 pandemic or lockdown. A baseline FSFI before the pandemic would have given a more objective basis for comparison of the FSFI score during the lockdown. The women in this study were highly educated, with >90% having tertiary education, yet the risk of recall bias cannot be overlooked. The perception/personal assessment of changes in their sexual function should have been assessed, and this may have been quite informative.

The impact of this study is that it provided a peek at the risk of FSD and the types of FSD in the study environment, putting into consideration the location and social, ethnic, and cultural variation of the people. It also gives an insight into the pattern of FSFI score among women in this environment. We recommend the use of the FSFI score with caution, including its interpretation in this population, as there is a lack of baseline data on FSFI score and a need for validation for the Nigerian population.

## Conclusion

FSD was prevalent, with the most common form being disorders of desire and arousal during the COVID-19 lockdown relative to the period before and after the lockdown. Women who tested positive for COVID-19 had a higher risk of sexual dysfunction during the lockdown. It would be interesting to further learn from women via a qualitative study to understand the deeper reasons behind their manifestations in order to design context-specific interventions for them.
